# Extreme Sub-Wavelength Structure Formation from Mid-IR Femtosecond Laser Interaction with Silicon

**DOI:** 10.3390/nano11051192

**Published:** 2021-04-30

**Authors:** Kevin Werner, Enam Chowdhury

**Affiliations:** 1 Department of Physics, The Ohio State University, Columbus, OH 43210, USA; kevin.werner@baesystems.com; 2 Department of Material Science and Engineering, The Ohio State University, Columbus, OH 43210, USA; 3 Department of Electrical and Computer Engineering, The Ohio State University, Columbus, OH 43210, USA

**Keywords:** laser induced periodic surface structure (LIPSS), mid-IR, femtosecond laser, surface structuring, nano-structure, ultrafast melting, laser induced damage, ATG instability, surface engineering

## Abstract

Mid-infrared (MIR) wavelengths (2–10 μm) open up a new paradigm for femtosecond laser–solid interactions. On a fundamental level, compared to the ubiquitous near-IR (NIR) or visible (VIS) laser interactions, MIR photon energies render semiconductors to behave like high bandgap materials, while driving conduction band electrons harder due to the λ${}^{2}$ scaling of the ponderomotive energy. From an applications perspective, many VIS/NIR opaque materials are transparent for MIR. This allows sub-surface modifications for waveguide writing while simultaneously extending interactions to higher order processes. Here, we present the formation of an extreme sub-wavelength structure formation (∼λ/100) on a single crystal silicon surface by a 3600 nm MIR femtosecond laser with a pulse duration of 200 fs. The 50–100 nm linear structures were aligned parallel to the laser polarization direction with a quasi-periodicity of 700 nm. The dependence of the structure on the native oxide, laser pulse number, and polarization were studied. The properties of the structures were studied using scanning electron microscopy (SEM), atomic force microscopy (AFM), cross-sectional transmission electron-microscopy (CS-TEM), electron diffraction (ED), and energy-dispersive X-ray spectroscopy (EDX). As traditional models for the formation of laser induced periodic surface structure do not explain this structure formation, new theoretical efforts are needed.

## 1. Introduction

Strong field interaction with solids in the mid-infrared (MIR) regime of light provides an exciting platform in contrast with that of visible (VIS) and near-infrared (NIR) regimes [1,2]: first, the reduction of photon energy allows the interaction to be more field dominated than photon dominated, where the material valence band to conduction band electronic transition leaning away from multiphoton processes towards tunneling process; second, traditional semiconductors and other ’small bandgap’ materials, which are highly absorptive in the VIS-NIR regime, become transparent in the MIR, potentially changing ultrafast absorption dynamics; third, cycle averaged energy (a.k.a. ponderomotive energy, $U_{p}=e^{2}E_{0}^{2}\lambda^{2}/4m_{cb}$, where λ is the laser wavelength, $E_{0}$ is the peak laser electric field strength, and $m_{cb}$ is the effective mass in the conduction band) of the free carriers increases significantly with longer wavelength, changing free carrier absorption and collisional ionization; fourthly, with decreasing plasma critical density as wavelength increases, semiconductors may become ideal plasmonic platforms, creating exotic metamaterials and surfaces [3]. With the vast array of important applications available with controlled laser induced structure formation demonstrated with shorter wavelengths, e.g., solar cells [4], black silicon [5], waveguide fabrication [6], surface-enhanced Raman scattering [7], colorization [8], fabrication of hydrophobic surfaces [9], and many others, there is a lack of both experimental studies and theoretical considerations on this topic in the MIR regime.

With transparency extending to λ = 5 μm, silicon is an important MIR material with, by far, the widest industrial platform to date. It has also been used as a fundamental platform for the study of laser induced periodic structure (LIPSS) formation [10] and its many applications [11]. Multipulse LID effects have been studied extensively on silicon with NIR wavelengths [12]. One of the first extensive femtosecond LIPSS studies on Si revealed fine ripples, also known as high-spatial frequency (HSFL) LIPSS (period λ/4), along with coarse ripples, or low spatial frequency LIPSS (LSFL) (period 0.8λ) in the NIR regime (λ=800 nm, pulse duration = 100 fs, and 0${}^{\circ }$ incidence, AOI) on *p*-doped (100) silicon [13], under vacuum with oxide layer etched. HSFL was observed to be aligned to the electric field polarization, indicating a surface scattered wave (SSW) mechanism [14], while LSFL was perpendicular to the electric field polarization, pointing towards a surface plasmon polariton (SPP) mechanism [15]. Under circular polarization, straight ripple patterns can still form but require a larger number of pulses [16].

LSFL has also been observed in various works on Si in the NIR regime (e.g., [17,18,19], without any evidence for HSFL formation. It has also been shown that LIPSS formation happens on the same timescale as material removal, suggesting that material is ejected [19]. An earlier study in the short wave IR (SWIR) regime (1300–2100 nm) showed that, beyond NIR, multi-pulse femtosecond interaction with Si produces not only traditional LSFL in the central region of the damage spot, but also a different type of HSFL feature with a period as small as λ/6 [20]. LIPSS formation on silicon has also been studied using ultraviolet (UV) wavelengths. A recent paper reports nano-island and HSFL formation on lightly doped n-type Si (100) at 0${}^{\circ }$ AOI with 390 nm wavelength, and 150 fs pulse duration under rough vacuum. The authors observe within a single shot: nano-islands, bifurcated islands, HSFL, and LSFL, with increasing local fluence, respectively. This indicates a universal origin for HSFL and LSFL [21].

Determinations of the multi-shot laser-induced damage threshold (LIDT) of silicon with NIR wavelengths have also been well studied. Experiments have considered variations in the pulse duration [22,23], as well as number of pulses [24,25].

While studies of this sort using MIR wavelengths on silicon are generally lacking, LIPSS formation has been studied on other materials with MIR wavelengths. For example, studies in the picosecond regime on GaP and CaF${}_{2}$ have observed damage initiated from surface defects, including LSFL [26], with λ=4.7μm laser wavelength and an LIPSS period of 2.5 μm. Several studies have been performed on Germanium with MIR wavelengths [27,28,29]. One such study observed HSFL both within a central damage spot and within a peripheral ring [29], where both types of HSFL were parallel to the laser polarization and the peripheral HSFL had double the spatial frequency as compared to central HSFL. The central frequency HSFL was about 0.27 times the wavelength for λ=3.6μm. Under cross-sectional transmission electron microscope (TEM), a thin 50 nm amorphous layer was found throughout a cross section of the HSFL. A very recent study of LIPSS formation on Si with MIR wavelength [30] (range λ = 2.5–4.5μm) reveals HSFL (λ/4) parallel and near-subwavelength LSFL perpendicular to MIR laser polarization, consistent with observation from past NIR experimental observations. In our present study, we present a systematic study of femtosecond laser surface structuring at a MIR wavelength, and show formation of completely new classes of surface structure formation, one extreme sub-wavelength (using linearly polarized pulses), and another one chiral (using circularly polarized pulses).

## 2. Materials and Methods

A home-built Ti:Sapphire ultrashort pulse laser [31] is converted to MIR wavelengths via the Extreme Mid-InfraRed (EMIR) optical parametric amplifier (OPA) [3,32] with central wavelength λ=3.6 µm, pulsed repetition frequency (PRF) of 500 Hz, and pulse duration of τ≈200 fs full-width at half-maximum (FWHM).

Using our previously-described laser-induced damage (LID) setup [1], high purity (>1000 Ω-cm) FZ mono-crystalline silicon (111) and (100) wafers were exposed to a maximum of 10,000 pulses per site with a maximum average peak fluence of 1.6 J/cm${}^{2}$, a focal diameter of 24 µm FWHM, at an angle of incidence (AOI) of 45 degrees (*p*-polarization). Details of the focal spot characterization method and positioning of the sample surface with respect to the focal plane are described in detail in reference [1]. The (111) wafer is oriented such that the laser pulse electric field polarization was parallel to the (100) direction. In this work, the reflex objective used for sample irradiation with MWIR pulses in reference [1] was replaced with an f=200 mm CaF${}_{2}$ lens. Each site was exposed to a predetermined number of pulses via a combination of the GRAY laser’s external Pockels Cell pulse-picker and a synchronized mechanical shutter. The average fluence of these pulses was controlled by a variable attenuator [1]. For some tests, a quarter waveplate (QWP) was inserted to study the effect of circular polarization.

All experiments were performed in air with air-exposed samples; a few nm thick native-oxide layers were expected to be present on the wafer surface. To determine the importance of the native oxide layer, some of the silicon (111) wafers were etched by a 10% hydrofluoric acid (HF) buffer solution. We estimate a maximum oxide-layer growth of <1 atomic layer based on a timed 8 min transfer from etching to irradiation and the average rate of oxide layer growth on silicon at room temperature (≤0.02 Å/min) [33].

Post-exposure examination of the samples was performed ex-situ via scanning electron microscopy (SEM), atomic force microscopy (AFM), cross-sectional transmission electron-microscopy (CS-TEM) [1], electron diffraction (ED), and energy-dispersive X-ray spectroscopy (EDX). CS-TEM, ED, and EDX were all performed on a single representative site in a representative region of the sample (500 pulses, 547 mJ/cm${}^{2}$ average peak fluence, (111) wafer, etched oxide layer).

## 3. Results and Discussion

LIDT fluence was determined for varying number of pulses. Here, we define damage as a permanent change induced by the laser as detectable by SEM.

**Figure 1:**
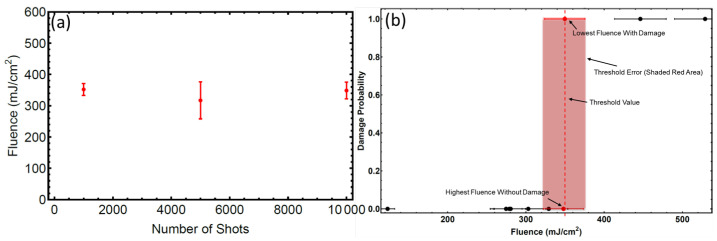
LIDT of silicon (111) vs. number of shots. (**a**) LIDT fluence vs. number of shots for Si; (**b**) example of damage threshold determination method for 10,000 shots. The LIDT here is considered to be the average of the highest fluence with zero damage probability and the lowest fluence with 100% damage probability.

The results of the LIDT study are shown in Figure 1. The S-on-1 multi-shot damage thresholds between 1000–10,000 shots were found to be shot number independent at around 350 mJ/cm${}^{2}$. This is consistent with a well-known phenomenon, where the material LIDT steadily exhibits reduction with increasing number of pulses so that F(infinity)/F(1) approaches an asymptotic value around F(1000) i.e., the 1000-on-1 damage threshold fluence [34]. The LIDT were determined by damage probability as described in Figure 1b. The LIDT error bar is considered to be within the range of possible fluence values for the highest fluence without damage and lowest fluence with 100% damage.

**Figure 2:**
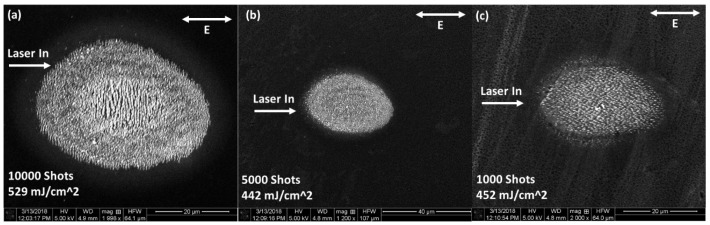
SEM micrographs of multi-pulse LID in silicon (111) with a high number of shots. From parts (**a**–**c**), the number of shots is decreasing while the fluence is mainly constant (up to fluctuations in the average pulse energy). Number of shots and fluence are located in the bottom left of the figure. In each figure, the input direction of the laser and polarization direction are indicated (as projected onto the sample surface).

Figure 2 shows SEM for a fixed fluence with 10,000, 5000, and 1000 shots. With 10,000 shots, central LSFL and peripheral HSFL are observed. In both cases, the structures are elongated in a direction perpendicular to the electric field polarization.

Decreasing the number of shots to 5000; the LIPSS structures tend to disappear, resulting in chaotic damage features. Looking at 1000 shot damage sites, a new kind of structure begins to form in the peripheral region, predominantly on the side of the sample opposite to the laser input direction (i.e., on the right side of Figure 2c. These nano-structures will be the topic of focus for this study.

**Figure 3:**
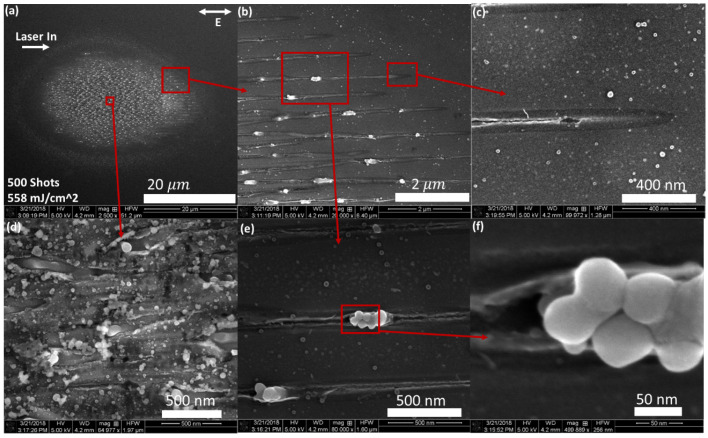
SEM images of multi-shot LID on silicon (111) with 500 shots. (**a**) shows an overall SEM image of the LID site. The fluence is indicated in the figure, as well as the direction of laser propagation and polarization, both projected onto the sample surface; (**b**,**d**) are zoom-ins of the red boxed areas in (**a**), as indicated by the red arrows; (**c**) is a zoom-in of a red boxed area in (**b**) as indicated by the red arrow; (**e**) is a zoom in of the larger red boxed area of (**b**), and an even larger magnification of (**e**) is shown in (**f**).

We found that the nanostructure formation works best with 500 pulse accumulated damage sites (see Figure 3). Two different types of nanostructures are observed. First, very narrow (<50 nm) regularly spaced (500 nm) nano-trenches extend out to the peripheral. The trenches are very straight and are parallel to the electric field polarization orientation. There appears to be a “rim” on either side of the trenches. Outside of the rim, there is a zone which has a contrast difference on the SEM. Drawing from the results of Ref. [1], this contrast change could be due to either subsurface melting or a weak ablation. Moving towards the center, the trenches bifurcate and become wider. Eventually they overlap and combine, resulting in apparent chaotic damage without a discernible structure. Debris can be seen around this area which may have come from the trenches.

The second type of observed nanostructure is a series of nanoscale outgrowths or clusters of “nano-spheroids”, as shown in Figure 3b,e,f. They are always found aligned to the trenches, consisting of multiple spheroids, and appear to be molten material that has erupted from the trenches. They are not as regularly spaced as the trenches, but they occur roughly every μm. The exact shape of the nano-spheroid clusters (NSC) vary, but they are found to be generally round, form in clusters which are around 200 nm long, with the width of an individual spheroid about 50 nm. Since these observations were from Si sample surface with native oxide layers present, an obvious question emerged: what is the role of the native oxide layer in the formation of these two types of nanostructures?

**Figure 4:**
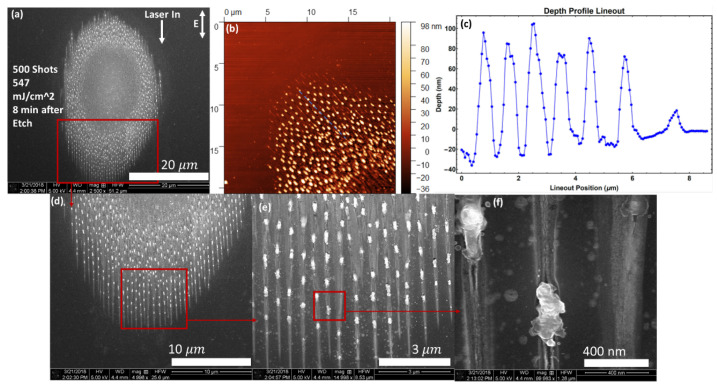
SEM and AFM images of multi-shot LID on silicon (111) with 500 shots, with oxide layer etched 8 min prior to exposure. (**a**) indicates the fluence used and the direction of laser input with polarization both projected onto the sample surface; (**b**) shows an AFM depth profile of the same sample area as in (**d**), but rotate by approx. 135 degrees clockwise. Samples with structures that may mimic AFM artifacts are purposely rotated to avoid scanning along or perpendicular to nanocracks; (**c**) lineout along the blue dotted line in (**b**); (**d**) shows a zoom-in of the boxed area in (**a**); (**e**,**f**) show further zoom-ins of this area indicated by the red boxes and arrows.

To test the effect of the absence of a native oxide layer, we used an etched silicon (111) sample (see the Methods section). The results are presented in Figure 4. The morphology in this case appears qualitatively different from those in Figure 3. Focus first on the trench structures, and it is observed that the width is 20–40 nm near an NSC and less than 10 nm away from them. The nanotrench width still increases towards the center of the damage spot, eventually merging with one another. The trenches in this case remain extremely straight “distinct” single lines over a much longer distance (nearly 10 μm) before bifurcating and merging into chaotic damage. The spacing between trenches is still about 500 nm at the peripheral. An AFM depth profile taken along the trenches show that the trenches may grow up to 40 nm deeper toward the center of the damage spot.

The nano-outgrowths also exhibit differences in the absence of the native oxide layer. While they are still spaced about 1 μm apart, their spacing is now more well defined and regular spacing occurs over the entire distance for which the trenches remain single lines. The shape of the nano-outgrowths is also different. Rather than distinct roughly sphere shaped clusters, we now see a single chaotic eruption of material without a well-defined shape. Furthermore, a closer look at Figure 4f shows that the debris in the area are also shaped differently. Instead of very round balls of debris, we see what appear to be disorderly clumps without definitive shapes.

**Figure 5:**
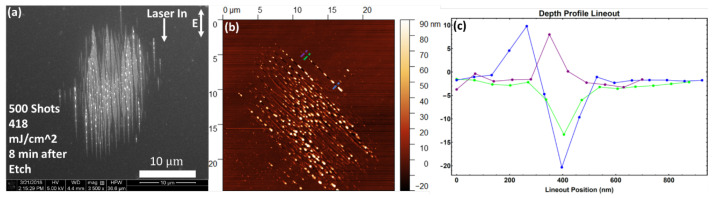
SEM and AFM images of multi-shot LID on silicon (111) with 500 shots, with oxide layer etched 8 min prior to exposure at a low fluence. (**a**) SEM image, indicates the fluence, laser input direction, and polarization projected onto the sample surface; (**b**) AFM depth profile, blue, green, and red dotted lines indicate lineouts in (**c**).

Under the same conditions (oxide layer etched, Si (111)), but with a lower fluence, the morphology changes (see Figure 5). In this case, the entire damage site looks more like the peripheral regions from the previous two figures. The trenches are still strongly aligned to the laser polarization and become wider towards the center. In addition, Figure 5c shows that the end of the trenches are raised relative to the original surface.

**Figure 6:**
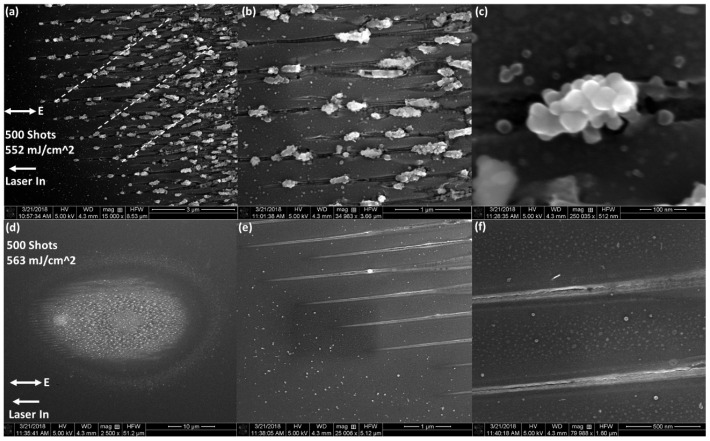
SEM images of multi-shot LID on silicon (100) with 500 shots. The laser input direction and polarization direction are indicated as projected onto the sample plane. The fluence is indicated on the bottom left for figures (**a**–**c**) and for figures (**d**–**f**); (**a**–**c**) show a damage site with progressively increasing magnification. The white dotted lines in (**a**) overlay ordering along a diagonal; (**d**–**f**) show a higher fluence damage site; (**d**,**f**) show increased magnification of the peripheral nano-trenches in (**d**).

Figure 6 shows the effect of switching to a (100) sample under similar laser conditions. Note that this sample was not etched to remove the oxide layer. Here, perhaps, there is some sign of a diagonal ordering of the NSCs across distinct nano-trenches. The sample was oriented such that the vertical and horizontal edges in Figure 6 are aligned to the (110) direction such that the diagonal ordering (white dotted line) was aligned to the (100) direction. The trenches seemed to bifurcate more readily along this diagonal direction. The trenches also seemed to have lost a well-defined ridge. The spacing between NSC eruptions had decreased to about 500 nm. These clusters of nano-spheroid outgrowths are less consistent. They showed a larger number of spheroids per clump.

Above a certain fluence (see Figure 6d–f), we found that the formation of the NSCs on the peripheral was suppressed while the trenches remain. Furthermore, at this higher fluence, the trenches have regained their ridges.

In order to obtain some insights into how the nanostructures formed, we looked at a higher fluence and varied the number of shots. A higher fluence was chosen so that the lower shot number exposures would still exhibit damage. Due to the Gaussian nature of the focal fluence profile on the surface, this allows us to explore features’ formation at several different fluences within the same site based on distances from the center of the site.

**Figure 7:**
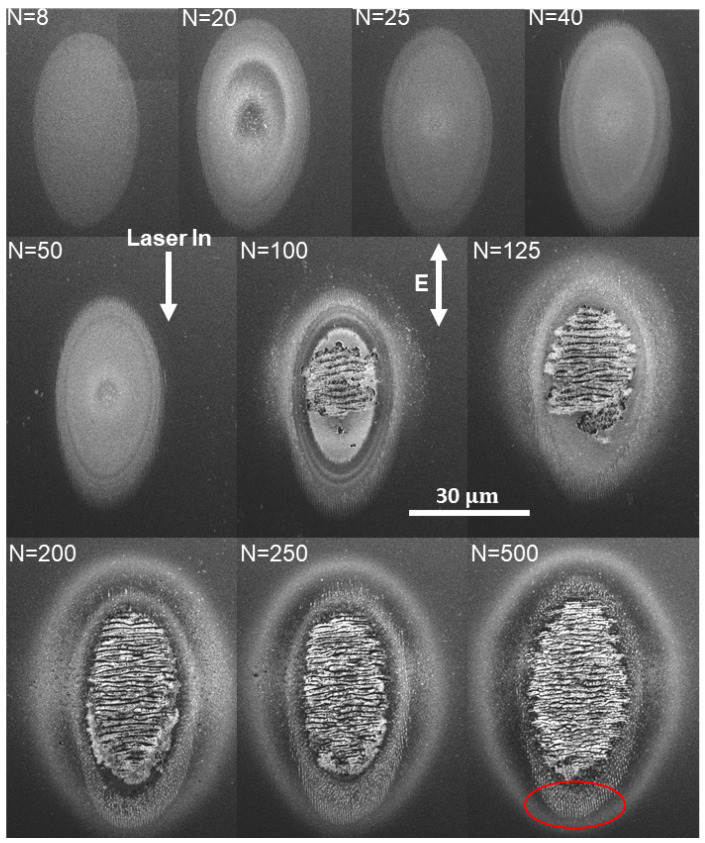
SEM images of multi-shot LID on silicon (111) with a fixed fluence and varying number of shots. The fluence used ranged from 0.8–0.9 J/cm${}^{2}$. The laser input and polarization directions are indicated as projected onto the sample surface. A universal length scale bar is indicated towards the middle of the figure. Each damage spot has the number of shots (N) indicated in the top left corner. The red circle in the bottom-right N=500 shots image shows the location where the nanostructures most strongly form with 500 shots.

The results of the shot number study are shown in Figure 7. At the site with 8 shot accumulation, we observe a damage area reminiscent of ultrafast melting/amorphization [24]. After 20 shots, one can observe what would eventually become the trenches, beginning to grow from the bottom rim of the site. The length of the trenches continues to grow until about 100 shots. At 100 shot accumulation and beyond, one observes a ’breaking’ of a top layer, revealing the wavelength scale LSFL growing underneath. Similar LSFL periods have been recently observed in Ref. [30]. 2D Fourier analysis of the sites from the last row (N= 200, 250, and 500 shots) do not reveal sharp periodicity. However, few distinct diffuse period peaks appear, and the strongest is a peak representing an average LSFL period of 1.4 μm, with a range from 0.6–2 μm. A weaker peak oriented the same way (perpendicular to the laser polarization direction) shows a smaller period of 0.37 μm. These smaller features could arise in two steps, first bifurcation of largest LSFLs resulting in 0.7 μm periods, and then a second bifurcation of these to obtain the smallest periodicities. The bifurcation mechanism may be similar to that observed in another semiconductor [35]. The nano-trenches forming in the peripheral region result in a periodicity of 0.5μm, as observed in previous figures. From sites with 125 to 500 shots, NSCs on nano-trenches begin to form in an increasing number corresponding to an increasing number of pulses per site.

Based on our observations so far, several characteristics begin to emerge pertaining to the formation of nano-trenches: first, they form via multi-pulse irradiation; second, their formation fluence is lower than multipulse ablation threshold, and the formation fluence range is relatively narrow; third, they do not form due to SPP driven mechanisms; fourth, it is unlikely that the surface scattered wave model applies to their formation mechanism, as their features appear qualitatively different from SSW HSFLs, which are also parallel to the laser polarization direction; fifth, the formation mechanism, albeit not needing the existence of native oxide, is nevertheless affected by the presence of native oxide on the surface, and perhaps the crystal orientation. We also note that some of the nano-structures presented here seem to resemble some of those presented in ref. [20], especially Figure 3 and Figure 5 there (generated by λ= 2100 nm) in comparison to our Figure 5. In both theirs and our cases, the photon energies were below the Si bandgap, theirs within two photon absorption regime, where ours were beyond three photon absorption regime. They used unetched (native oxide present on surface) doped n- and p-type Si samples, with dopant concentrations of $∼10^{15}$ cm${}^{-3}$, whereas, in our case, impurity concentrations were $∼10^{12}$ cm${}^{-3}$. Laser illumination was at normal incidence in their case, where we used 45 degree AOI at *p*-polarization, lowering Fresnel reflection in our case. Still, a comparison can be drawn between their “bump” period along a “line” of ∼1.1 μm (Figure 4 bottom panel), and ours from Figure 5c showing a period of ∼1 μm, which do not preserve wavelength scaling. On the other hand, for the “periods” of lines parallel to laser polarization direction, they showed two types, the first one presented in the Figure 4 top panel, with a period of of ∼900 nm, or λ/2.3 (“lines with bumps”), and the second ones at Figure 5 with spatial period of ∼370 nm (“lines without bumps”), or λ/5.7. In our case, lines (“nanocracks”) with or without bumps, parallel to laser polarization directions exhibit periods between 400–600 nm, i.e., between λ/9–λ/6.

**Figure 8:**
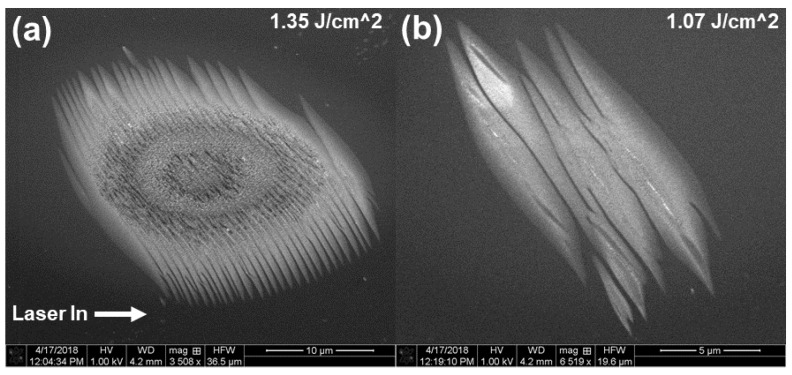
SEM images of multi-shot LID on silicon (111) with 500 shots and circular polarization. The fluence for each shot is indicated in the top right corner and the laser input direction is indicated in (**a**).

Next, focused circularly polarized MIR laser pulses were incident on (111) silicon by inserting a MIR quarter wave plate in the beamline before the focusing element. The results were quite surprising, with features qualitatively different than that from linear polarization case. SEM images of the damage sites generated by circularly polarized pulses is shown in Figure 8. Here, a higher fluence was used, since the damage threshold for circular polarization case was found to be generally higher.

A wavy, chiral pattern is observed in the damage sites. These waves appear to exhibit a contrast change in the SEM consistent with ultrafast melting and amorphization near the peripheral regions. The features narrow on the peripheral region. Trench like features can be seen towards the central region of damage, which exhibits multi-zone damage. The trenches seem to be aligned to the peripheral melting structures. A central region of damage seems to be surrounded by a ring of chaotic damage with no discernable structure aligned to the trenches. Lower fluence shots exhibit a similar wavy structure but without the trenches. The chiral nature of these surface features seem to imprint the handedness of polarization state (rotating clockwise) on these bright surface peripheral features, and may point towards an effect of strongly driven free carriers near the surface by the MIR electric field.

**Figure 9:**
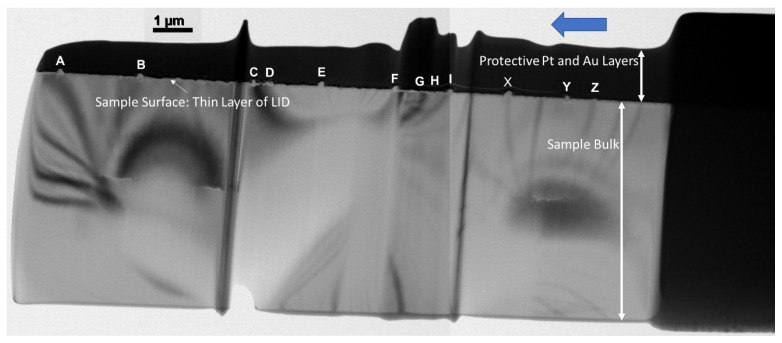
Cross-sectional TEM of a single, multi-shot LID site on silicon (111) with 500 shots, *p*-polarization. The sample oxide layer was etched 8 min prior to being shot by a train of pulses with an average fluence of 547 mJ/cm${}^{2}$. The large blue arrow points from the peripheral of the LID spot towards the center. This sample has undergone FIB processing as described in the Methods section. The letters indicate various points of interest. The different layers involved in the cross section are labeled in the figure. Additionally, a capital letter labels several areas of interest. They include raised features (NSCs or nano-spheroidal clusters) A–F,I,X,Y,Z and a few trenches G,H.

Cross-sectional transmission electron microscopy (TEM) was used to investigate one of the etched sample damage areas on silicon (111) with 500 shots and a fluence of 547 mJ/cm${}^{2}$. The results are shown in Figure 9. This figure is meant to familiarize the reader with the sample under low magnification. Uppercase letters mark certain locations of interest. Before moving forward, it is important to understand that this figure shows a cross section of the LID spot near the peripheral, along several holes and trenches. The largest features in the figure are the protective layers (Pt and Au) which appear as very dark, and the sample bulk, which has characteristic bend contours. Only a very thin region at the interface between the bulk and the protective layers has been modified due to the laser, barely visible in this image. This is why we present higher magnification TEM images next. Our objective is to investigate the structure of the sample underneath the surface: determine which parts are crystalline and amorphous.

**Figure 10:**
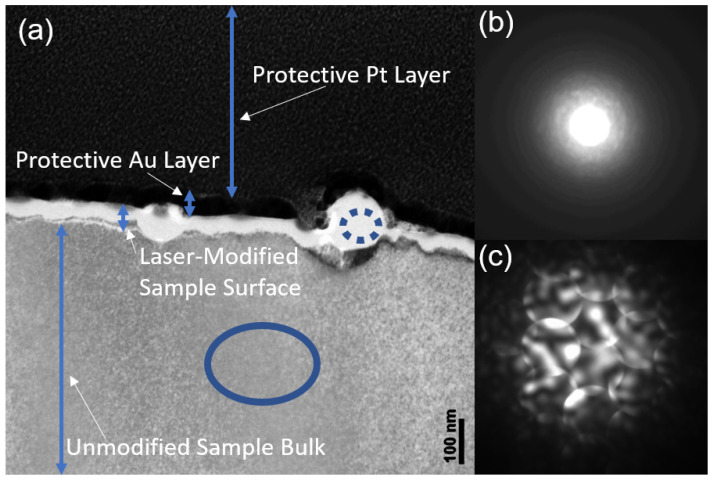
Electron diffraction and cross-sectional TEM measurements of multi-pulse LID on silicon. The different layers in the image are labeled in (**a**); (**b**) shows the result of electron diffractometry performed in areas like the blue dotted circle, i.e., on a nano-outgrowth; (**c**) shows the result of electron diffractometry performed in areas like the solid blue circle. It shows a strong crystalline ordering. In contrast, a strong amorphous signal is observed in (**b**). This means that, in the sample, the brighter white areas correspond to amorphized material while the gray/dark areas in the bulk correspond to monocrystalline silicon material.

Figure 10 shows a couple of the NSCs in higher magnification. The bright white areas correspond to amorphous material and gray corresponds to crystalline material, as evidenced by the ED scans in parts b and c. The TEM analysis shows that, away from NSCs, the laser modified surface amorphization is approximately 40 nm deep. Sometimes, black areas can be seen within the sample, attributed to voids or absence of material.

**Figure 11:**
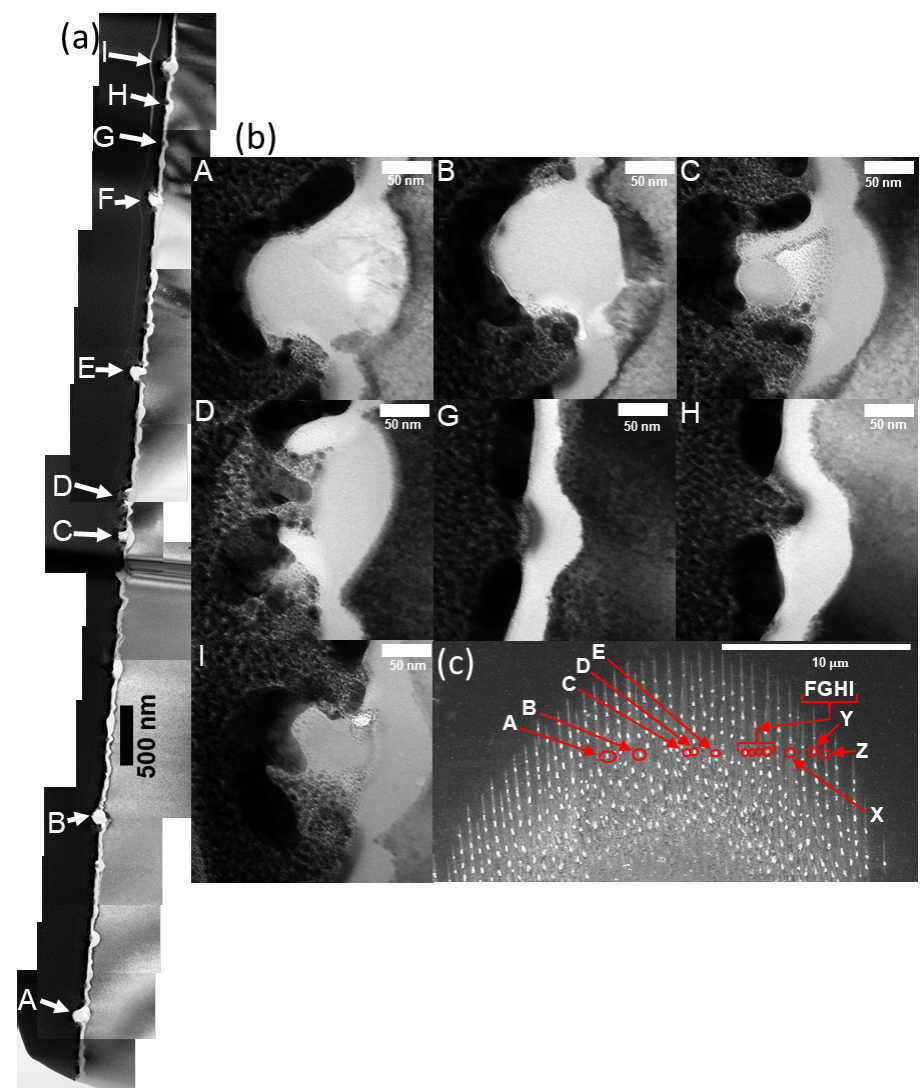
High resolution cross-sectional TEM measurements of multi-pulse nanostructure formation on silicon. (**a**) TEM images of a wide area have been stitched together. Areas of interest are marked by an upper-case letter. Area A is near the end of the sample (see Figure 5 and Figure 6); (**b**) several of the features from (**a**) are shown magnified. They are labeled by the corresponding capitol letter locator in the top left corner; (**c**) the sample location for each feature is labeled in an overhead SEM of the site taken prior to any FIB/TEM processing.

Figure 11 summarizes the results of the TEM study. At locations A and B, a bisection of a single NSC is captured. Clearly, the spheroids are amorphous and they sometimes have a void beneath them. At location C, a partially detached NSC can be seen. At location D, it seems an NSC has completely been detached, leaving behind a ridge-like feature. Locations A,B,C,D all have an NSC and an increase in the amorphization thickness underneath the nano-outgrowth or NSC. Location G and H show 20–50 nm trenches. Clearly, the amorphous layer is thicker beneath the trenches, but rather than an NSC, there is an absence of material (which points towards crack formation). Location I shows another NSC. A slight contrast change can be seen beneath the NSC, which could indicate that the material there is at a lower density, i.e., the NSC may have been starting to detach as it cooled.

Next, we will explore the elemental make-up of the NSCs and surrounding material. In order to investigate this, we performed EDX analysis on the NSC at location Y.

**Figure 12:**
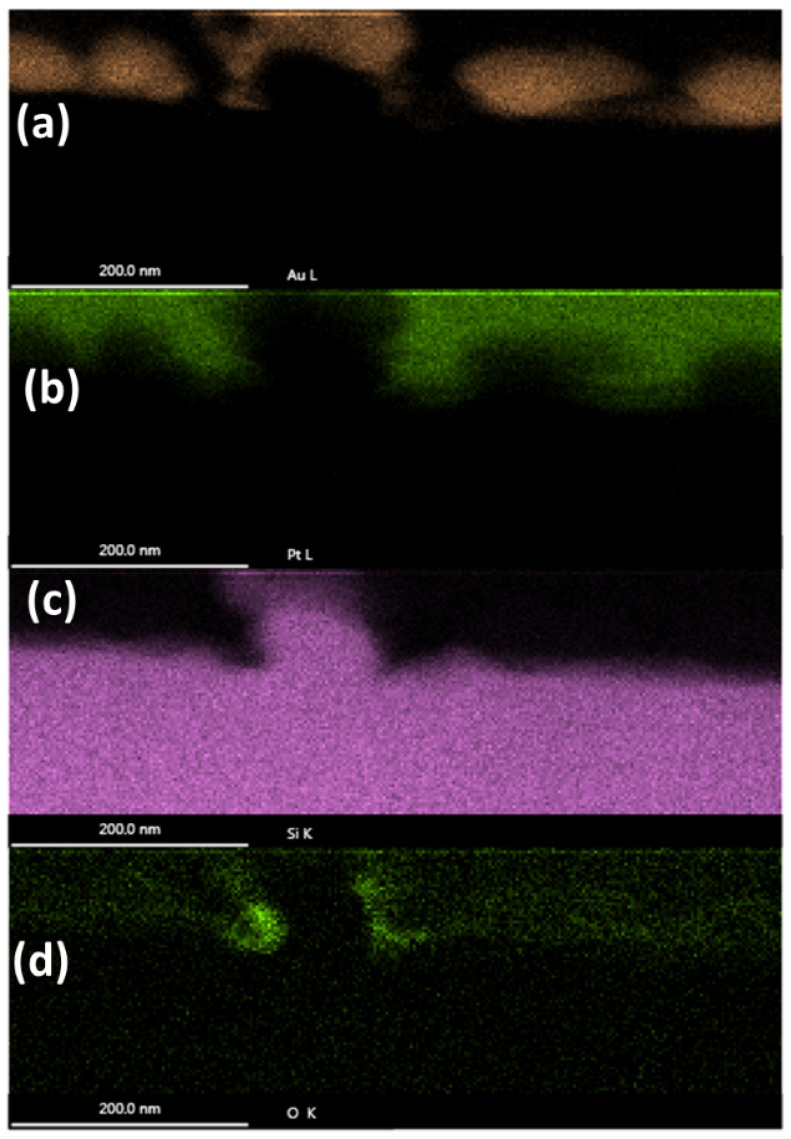
Spatially resolved Energy-dispersive X-ray spectroscopy (EDX) of an NSC outgrowth. The EDX is taken at location Y of the cross-sectional TEM sample. The peak signal count value is plotted for Au (**a**), Pt (**b**), Si (**c**), and O (**d**).

The results of the EDX study are shown in Figure 12. The peak value of four different elements is displayed for each location by the intensity of the pixel at that location. The gold from the protective layer, which clusters together, can be seen clearly above the surface. Pt clearly has been deposited above the sample everywhere besides where there is gold. The silicon signal is strongly distinguishable, although near the top of the NSC, it seems some gold has penetrated. Finally, significant oxygen is only seen near the undercut of the NSC.

**Figure 13:**
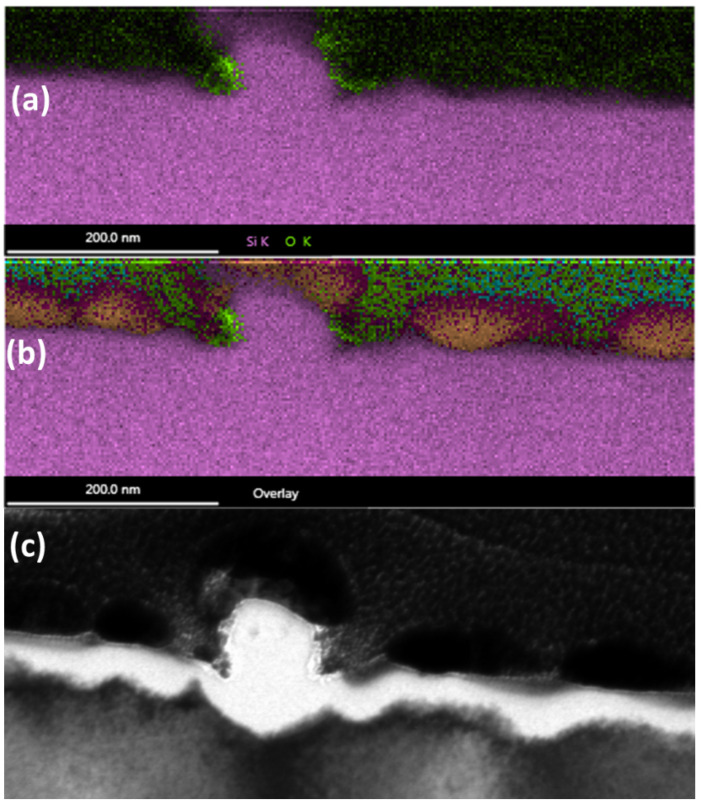
Spatially resolved Energy-dispersive X-ray spectroscopy (EDX) of an NSC outgrowth combining elemental signals. (**a**) silicon and oxygen peaks plotted; (**b**) Silicon, Copper, Gallium, Platinum, Gold, Carbon, and Oxygen are all plotted together, as color coded by the words used to describe them here; (**c**) cross-sectional TEM of the area which has undergone EDX in (**a**,**b**).

A proprietary algorithm used by the EDX software is able to plot spatially resolved EDX of several elements simultaneously while allowing for the colors to be mixed if both elements are present at the same location. This is shown in Figure 13.

Figure 13 clearly indicates that the NSC outgrowth is not made of a silicon-oxide. Instead, it is made of pure silicon. Furthermore, the top part of the NSC was contaminated by gold during FIB preparation. Near the undercut of the NSC, higher levels of oxygen are indeed present. The very thin, brighter white area in Figure 13c is characteristic of an oxide layer. Carbon is expected to be present on the sample since it was exposed to air. Gallium is present from the gallium FIB. The Cu signal is from scattering off of the copper sample mount.

Based on our analysis of these unique extreme sub-wavelength surface structures (smallest nano-trench size 20 nm =λ/180) in single crystal Si generated from multi-pulse femtosecond MIR pulses, it is clear that the formation mechanism of these nanotrenches do not correspond to traditional LIPSS forming mechanisms. As it is clear from Figure 3, Figure 4 and Figure 5 that these straight-line like structures are formed by cracks on the surface, one may consider a very well known ATG instability theory, named after pioneering works of Asaro, Tiller [36], and Grinfeld [37]. The ATG model has been successfully used to explain many different types of periodic surface structure generation caused by induced stress in the surface layer [38,39,40]. The basic mechanism of such a stress induced periodic surface structure formation (SIPSS) in creating self organized nano-islands [41] and nano-ripples [42] is explained briefly thus: a flat surface under stress can relax its strain energy by undulating the surface via surface diffusion, which may also result in crack formation. For SIPSS, the stress is introduced by lattice mismatch between the surface solid solution layer and the substrate, whereas, in the case of the nano-trenches in discussion here, the stress is essentially localized ’thermal stress’ on the surface layer introduced by the laser. The strain energy is then relaxed via a process similar to the ATG instability, thus creating periodic trench/crack structures on the surface. There are two previous observations that support the above-mentioned hypothesis. First, these structures can only be generated after multiple laser shots. This can be explained by the fact that multiple laser shots induce more defect states, and disorder into the material, thereby increasing the strain energy. The sign of laser energy deposition and extreme disordering at the surface is also apparent due to the formation of the ∼40 nm deep amorphous Si (a-Si) layer at the top (see Figure 13c), which contains all the nano-cracks and NSCs. On top of that, a-Si is in a metastable state, and thus always subject to local structural rearrangements when perturbed by external energy input, in an effort to minimize free energy. With a lower melting point of ∼1200 ${}^{\circ }$C, a-Si also exhibits a significantly larger increase in absorption co-efficient in comparison to crystalline Si [43]. The heat generated by the laser (via free carrier absorption on the surface by the laser E-field parallel to the surface) can also enhance mass transfer via surface diffusion in the top layer. Mass transfer and diffusion towards the surface are also corroborated by the nano-spheroid clusters of pure a-Si (see Figure 12) appearing to be ’bursting’ out of the trenches/cracks in near-periodic fashion. The amorphous nature of Si NSCs also points towards ultrafast melting to locally reach temperatures above the Si melting point of 1410 ${}^{\circ }$C, followed by rapid cooling to prevent recrystallization. Meanwhile, a recent work on bifurcation of LIPSS on GaAs [35] shows that LIPSS bifurcation crack formation due to stress on the surface created by diffusion of defects towards surface do not occur until hours after the laser treatment, whereas the plasmon should immediately die away upon laser cut-off. This time-dependent phenomenon may be explained by slow kinetics of surface diffusion at low temperatures, which may take seconds to hours for the strain energy to relax. We plan to study the formation time scale of such structures in a future effort.

## 4. Conclusions

In summary, we have observed a new kind of ’LIPSS’ generated by strong MIR field interaction with single crystal Si with several distinctive features compared to traditional HSFL and LSFL. These ’straight-line’ Nano-cracks/trenches were aligned to the laser polarization forming a near-periodic structure on the peripheral of the damage spot, with a spatial period similar to HSFL formed on germanium with MIR wavelengths [29]. However, unlike the case for common HSFL, the size/width of the individual features which make up the periodic surface structure is much smaller than both the spatial periodicity of the lines and the laser wavelength (as small as λ/180), and this size depends strongly on distance from the central damage site. Furthermore, nano-spheroidal cluster outgrowths of molten silicon have been captured re-solidified in their final amorphous state; in some cases, these nano-spheroidal clusters or NSCs, were in the middle of escaping the material. To the best of our knowledge, nothing like this has been previously reported. This phenomenon occurs regardless of the presence of a native oxide layer, although the morphology is slightly different. The morphology of the structures also was found to be affected by the crystal orientation of the sample. Silicon oxide was not detectable within the nano-cracks or NSCs, although an increased presence of oxygen was found in the undercut of the NSCs. Further theoretical models will be needed to understand how these structures form. With more study, better control over the formation of these structures could lead to extremely straight, nanometer sized extreme-sub-wavelength (ESW) nanostructure device formation.

## Data Availability

Data can be made available by the authors upon request.
